# Mapping urban mobility using vehicle telematics to understand driving behaviour

**DOI:** 10.1038/s41598-024-53717-6

**Published:** 2024-02-08

**Authors:** Junjun Xiang, Omid Ghaffarpasand, Francis D. Pope

**Affiliations:** School of Geography, Earth, and Environmental Sciences, University of Birmingham, Birmingham, UK

**Keywords:** Environmental impact, Civil engineering

## Abstract

Telematics data, primarily collected from on-board vehicle devices (OBDs), has been utilised in this study to generate a thorough understanding of driving behaviour. The urban case study area is the large metropolitan region of the West Midlands, UK, but the approach is generalizable and translatable to other global urban regions. The new approach of GeoSpatial and Temporal Mapping of Urban Mobility (GeoSTMUM) is used to convert telematics data into driving metrics, including the relative time the vehicle fleet spends idling, cruising, accelerating, and decelerating. The telematics data is also used to parameterize driving volatility and aggressiveness, which are key factors within road safety, which is a global issue. Two approaches to defining aggressive driving are applied and assessed, they are vehicle jerk (the second derivative of vehicle speed), and the profile of speed versus acceleration/deceleration. The telematics-based approach has a very high spatial resolution (15–150 m) and temporal resolution (2 h), which can be used to develop more accurate driving cycles. The approach allows for the determination of road segments with the highest potential for aggressive driving and highlights where additional safety measures could beneficially be adopted. Results highlight the strong correlation between vehicle road occupancy and aggressive driving.

## Introduction

Road transport is a core component of the modern world and is closely linked to development and prosperity. For example, road transport connects workers to jobs, students to schools, patients to hospitals, and manufacturers to markets. However, it has also been linked to adverse and unsustainable consequences such as air pollution^1^, climate change^2^, traffic noise^3^ and road accidents. The most recent report from the World Health Organization^4^ on global road safety reveals alarming statistics regarding road incidents. It indicates that road incidents continue to be the leading cause of death for individuals aged 5–29, with nearly 1.2 million deaths annually, equating to one person dying in a road accident every 25 s. The economic impact of road incidents is estimated to be $1.8 trillion annually, which accounts for approximately 10–12% of global gross domestic product (GDP)^4^.

Human factors remain a major influence upon almost all the unsustainable dimensions of road transport^5^. Within sustainable transportation studies, the human factor is usually referred to as driving behaviour, and is determined using either kinematics or contextual approaches^6^. While the former is an aggregate approach to investigate the driving behaviour of a population of vehicles, the latter deals with the driving styles, actions, reactions, and individual driver responses. Driving behaviour studies (kinematics and/or contextual) are constructed on the analysis of the driving dynamics on the roads, see for example Wang et al.^7^. Driving dynamics estimated by vehicle speed-time variation characteristics can also provide an accurate picture of driving style in terms of aggressive or calm (relaxed) driving^8^, as well as vehicular fuel consumption and emissions^9,10^.

Contextual studies of driving behaviour can be traced back to the 1950s when McFarland^11^ conducted a research programme for highway safety using observers and in-vehicle instruments. Since then, civilian access to global positioning systems (GPS) has resulted in a surge in the amount of location data collected from roads. Consequently, a significant amount of research has been dedicated to investigating contextual factors influencing driving behaviour, see the review by Du et al.^12^. The substantial amount of collected data supports the application of advanced technologies in the prediction of aggressive driving behaviour. For example, Zahid et al.^13^ and Xu et al.^14^ utilised machine learning algorithms to predict aggressive driving among the general public and taxi drivers, respectively.

As previously discussed, contextual studies offer valuable information on individual driving patterns. Nonetheless, studying the behaviour of a vehicle population through a kinematic approach is vital for transportation, urban geography, and urban mobility research. For example, the contextual research offers an in-depth comprehension of driver intent in aggressive driving^14^ or female/male driving offenders’ behaviour^15^, but it overlooks the spatiotemporal examination of aggressive driving within the urban areas or the contribution of sociodemographic factors in shaping hotspots of aggressive driving.

The earliest investigations into driving behaviour from a kinematic standpoint were built upon the development of driving cycles (DCs). DCs provide speed-time profiles, which represent the driving pattern of a population of vehicles moving in a certain region. Several national and international DC protocols have been developed^16^. DCs were introduced in the transport sector almost four decades ago, see the literature review of Ghaffarpasand et al.^17^. They are tools utilised by car manufacturers to provide a long-term basis for design, tooling, and marketing. They also provide details on driving behaviour characteristics such as the percentage of journey spent idling, cruising, accelerating, or decelerating. DCs can also be used to study the influence of socio-demographics upon road transport. For example, Ghaffarpasand et al.^17^ analysed the DC of a city in Iran, namely Isfahan, to discuss the potential effects of public culture and urban development on the driving behaviour and emissions of moving vehicles. The results show that unsustainable development increases vehicle emissions as well as aggressive driving.

Despite such significant applications, DCs face several technical challenges. For example, DCs are usually developed from a small number (typically less than five) of GPS-connected vehicles travelling on specific routes of the study area through pre-designed programs, see for example Ghaffarpasand et al.^18^. Hence, DCs typically have coarse temporal and spatial resolution, which only encompasses the yearly transport situation of a city-scale region. Such limitations in data sampling indicate that DCs can only provide a snapshot of urban transportation and cannot capture the full complex dynamics of driving within the urban environments.

There are many efforts in the literature to analyse vehicle dynamics and thus driving behaviour with better spatial and temporal resolutions using vehicle GPS data. For example, Eboli et al.^19^ used data collected from the smartphones of 13 drivers to classify the driving behaviour of driving on a 138 km long road in the Calabria region, in southern Italy. However, the aforementioned research did not offer a comprehensive analysis of urban driving behaviour concerning temporal factors like differences between weekdays and weekends or rush and non-rush hours. Many of the studies aggregated and analysed road data collected over a prolonged period (such as nine year^20^), and subsequently investigated driving behaviour within certain locations. The representation of driving behaviour through snapshots proves inadequate in capturing the ever-changing dynamics of the urban environment. The absence of substantial spatial and temporal road data noticeably contributes to the inconsistency of the results.

Vehicle telematics data can address these drawbacks. Although the names telematics and GPS are used interchangeably, vehicle telematics data typically refers to data collected from a population of vehicles for specific purposes such as freight logistic support, driving behaviour assessments, fleet control/management, etc. The primary origin of telematics data comes from the on-board vehicle devices (OBD) fitted in cars where drivers strive for fair insurance premiums and, therefore, voluntarily reveal location information to telematics/insurance providers^4^. There has also been a considerable increase in the collection of vehicle telematics data via the driver's mobile phone in recent years. In 2017, it was estimated that there were more than one million live GPS-connected vehicles on UK roads^21^.

The utilisation of telematics data in studies of contextual driving behaviour has been extensively documented in the literature. For example, Ayuso et al.^22^ used telematics data (collected from the OBDs) to discuss the effect of the distance travelled on crash risk among young drivers. Their results indicated that gender has a notable effect upon the time of the first accident. The effect of distance travelled and the exposure time was also analysed by Boucher et al.^23^ using OBD telematics data, whereby their results prove that these factors should be involved in the driving behaviour rating system. Ayuso et al.^24^ and Guillen et al.^25^ consider these characteristics in addition to other risk factors such as the percentage of distance travelled over the speed limits, in urban areas and during the night to evaluate the driver’s contribution to traffic accidents. Results of Ayuso et al.^24^ show that driver habits can significantly affect the expected number of accidents. The wide applications and limitations of vehicle telematics data in road contextual driving behaviour studies and road safety are reviewed by Ziakopoulos et al.^26^ and Ghaffarpasand et al.^21^.

The potential for enhancing comprehension of contextual driving behaviour via vehicle telematics data is promising. However, further exploration of the use of telematics data in researching kinematic driving behaviour is needed as current studies are limited in number. Fu et al.^20^ used nine years of driving data collected from highways in Ann Arbor, Michigan, US, to construct spatiotemporal driving volatility profiles. A significant portion of the data was obtained from OBDs. They defined the concept of driving volatility as the percentage of non-majority driving instances. They did not use road data to analyse urban journeys. Instead, they followed the conceptual framework proposed by Liu and Khattak^27^ to process historical data and update it with real-time data. The study did not furnish details of driving behaviour characteristics and temporal evaluations over different hours of the day and days of the week. However, the results showed a strong correlation between driving volatility with the spatial and temporal occurrence of historical traffic accidents. Alrassy et al.^28^ analysed location data obtained from the OBDs of 4000 vehicles in New York City, US, between 2015 and 2016. The authors extracted driving behaviour indices including average speed, speed variation, hard braking rate, and hard acceleration rate, for specific road segments. These indices were then compared with the frequency and rate of crashes at the street level. The findings highlight the considerable potential of GPS-data-based methodologies to enhance further the present comprehension of road safety evaluation. However, the estimation of driver behaviour indices for each road segment was based on 1.5 years of extensive telematics data. Therefore, the study neglected the temporal variations, dynamic nature of the driving behaviour characteristics, and spatiotemporal distribution of aggressive driving in urban environments.

In this study, we propose a new approach to convert vehicle telematics data into driving behaviour characteristics including volatility. We then discuss the impacts of temporal and spatial factors upon these characteristics. We use comprehensive telematics datasets collected from the roads of West Midlands, UK, for the years 2016 and 2018 as the raw material.

## Results

### Driving behaviour characteristics examined by this study and the developed DCs around the world

**Figure 1:**
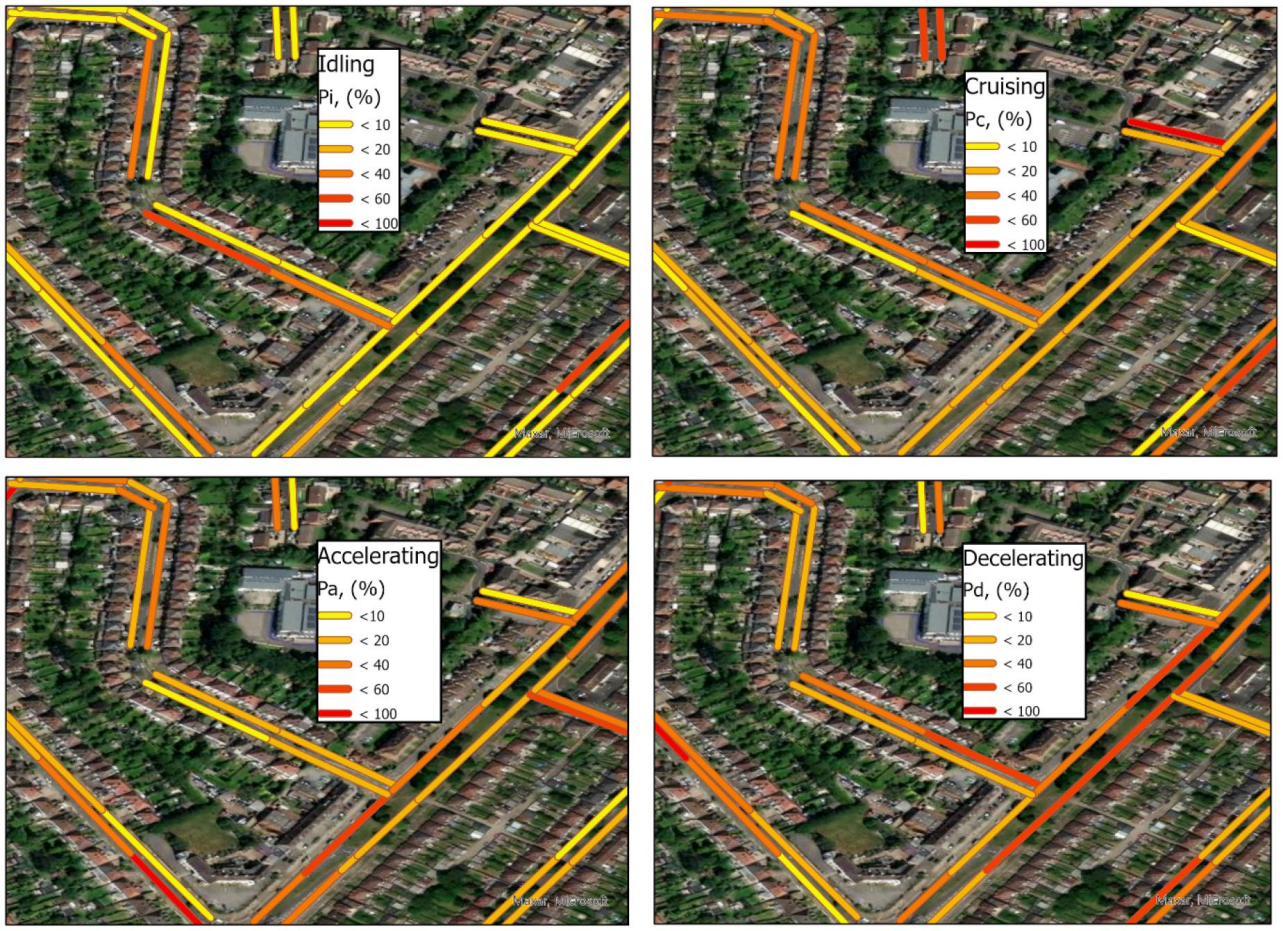
The spatial distribution of the driving behaviour characteristics over a small area of the city of Birmingham in the UK for Mondays 16:00–19:00 2018. The labelling of spatial characteristics of the maps have been removed for the rationale given in the text.

The new methodology introduced in this study (see “Materials and methods” section) provides a spatiotemporal mapping of driving behaviour characteristics across urban environments. For example, in Fig. 1, we present the spatial distribution of the different contributions of idling, cruising, acceleration and deceleration states over a small area within the city of Birmingham for Mondays 16:00–19:00 2018. Note that the labelling of spatial features within the maps has been removed to avoid any negative (or even positive) impacts on local businesses and residents.

As previously mentioned, the existing DCs worldwide usually have coarse spatial and temporal resolutions, offering road transport annual status within city-scale regions. However, the proposed methodology in this study can estimate the driving behaviour characteristics using fine spatial and temporal resolutions of 15–150 m and 2 h, respectively, when compared to the typical DC resolutions.

The driving behaviour characteristics estimated here and extracted by the DCs developed around the world are reported in Table 1. Driving behaviour can be determined by dynamic and static behaviour modes, whereby acceleration and deceleration status are dynamic driving behaviour modes and cruising, and idling statuses are the static mode of driving. Table 1 shows that driving behaviour across the West Midlands has almost 60% and 40% driving in dynamic and static modes, respectively. Similar contributions are currently estimated for accelerating and decelerating on the one hand and cruising and idling on the other hand.

**Table 1:** Driving behaviour characteristics estimated here and by DCs developed around the world.

DC	Area	Pi (%)	Pa (%)	Pd (%)	Pc (%)	Average speed (km/h)	Travel time (s)	Ref.
This study, 2016	West Midland, UK	21.6	28.5	28.4	21.5	24.1	NA	
This study, 2018	West Midland, UK	22.1	29.3	29.7	19.0	24.5	NA	
NEDC	European Union	20.4	23.6	17.3	38.8	42.2	965	^44^
ECE15 + EUDC	European Union	20.9	27.1	19.8	32.2	41.1	939	^44^
FTP-72	Australia	13.8	37.0	31.2	18.0	36.6	1180	^44^
FTP-75	USA	12.9	36.5	30.6	20.1	39.2	1633	^44^
NYCC	New York, USA	31.1	29.4	29.3	10.2	16.6	412	^44^
Unified LA92	Los Angeles, US	12.3	40.9	35.5	11.3	45.2	1258	^44^
JP 10–15	Japan	26.1	29.6	26.2	18.2	30.7	120	^44^
ISFDC	Isfahan, Iran	19.8	32.2	34.7	12.1	26.7	1863	^17^
CLTC	China	22.1	28.6	26.4	22.8	29.0	1800	^30^
WLTC	Worldwide	12.7	30.9	28.6	27.8	46.4	1800	^45^

In Table 1, we compare the driving characteristics estimated here with those estimated by different driving cycles (DCs) developed around the world. It is noted that the West Midlands, the study location for this study, contains both urban and extra urban features, but it is predominantly urban in character. The New European Driving Cycle (NEDC) is perhaps the most famous DC and is used by many government and private sectors around the world to assess vehicle emissions. The NEDC, developed in 1992, is based upon theoretical driving profiles, and consists of two separate parts for model driving over urban and extra-urban road conditions. In 2017, the Worldwide Harmonised Light Vehicles Test Procedures (WLTP) was introduced to replace NEDC, although NEDC is still used in many parts of the world. WLTP is longer (in terms of driving time) and more representative of real-world driving. The ECE + EUDC test cycle is generally used for European-type approval of light-duty vehicles to determine emissions and fuel consumption, while the Federal Test Procedures (FTPs) were introduced by the US Environmental Protection Agency (EPA) and are mainly used in North America. The reader is invited to read the review article of Huang et al.^29^ for more information.

Almost all DCs around the world have been developed for a specific driving time (see Table 1 for example), so the driving characteristics have been examined for the constant time intervals. In this study, however, the driving characteristics were examined by annual averaging. Therefore, different driving characteristics are reported for the study years (2016 and 2018).

The contribution of dynamic driving behaviour mode in NEDC and ECE15 + EUDC is 40–47%, the lowest reported here. The contributions of the dynamic mode of driving for the US, China, Japan, and Worldwide are near 67%, 54%, 56%, and 58%, respectively.

A high level of similarity is observed between driving behaviour characteristics estimated here and by the China Light-duty vehicle Test Cycle (CLTC). CLTC was developed by Liu et al.^30^ using real-world driving data collected from the roads of 70 cities across China. It was developed by the largest and most extensive dataset in DC development worldwide. Data from 3767 test vehicles were used, while most of the existing DCs are developed by the data from multiple cars. The low average speed of CLTS compared to that of famous DCs such as FTP-72, FTP-75, NEDC, and ECE15 + EUDC was also demonstrated by Liu et al.^30^. The calculated average speed here is currently close to the value estimated for CLTC and is the lowest among other well-known DCs worldwide.

### Driving behaviour characteristics over time

**Figure 2:**
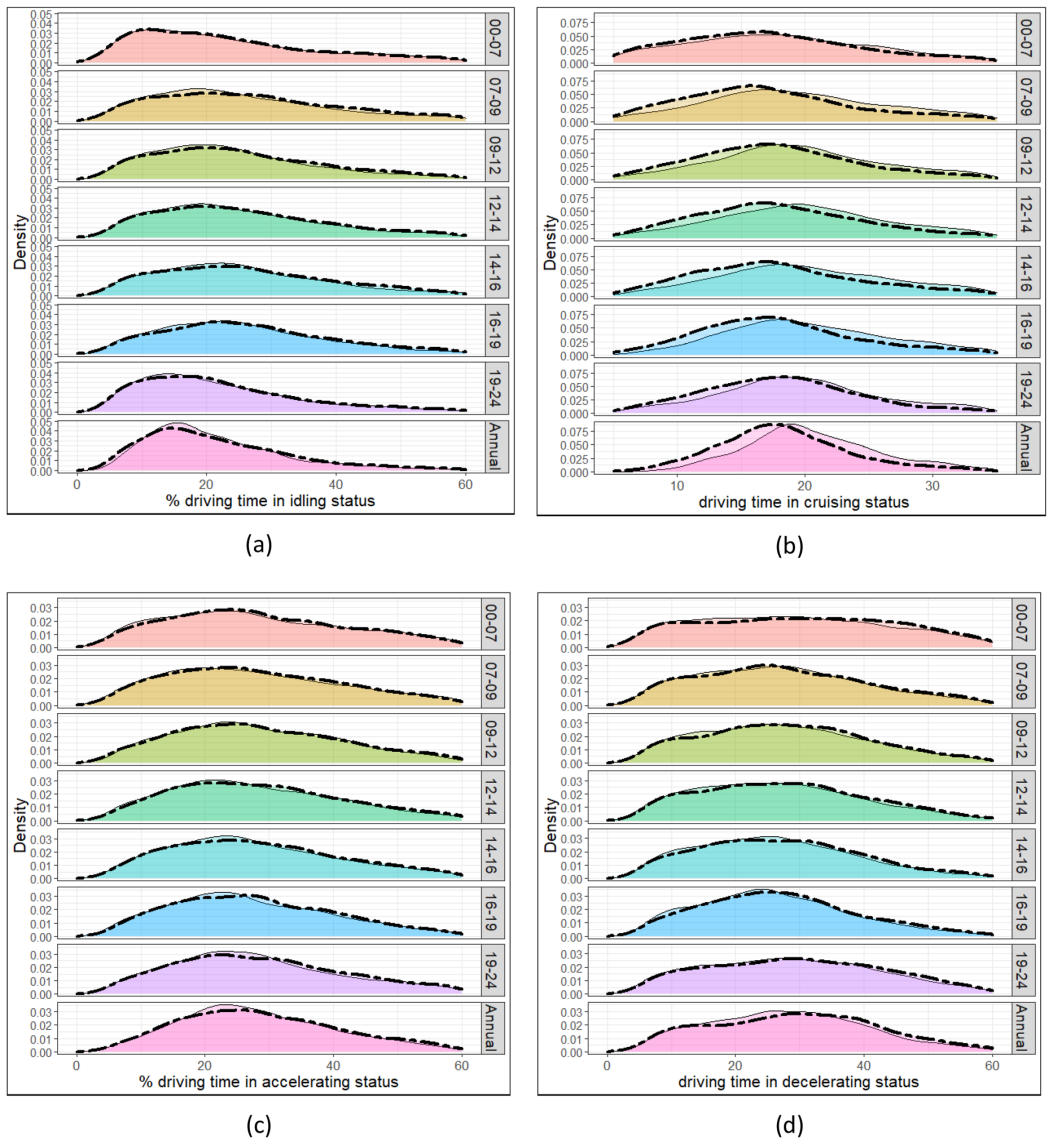
The probability distribution function (PDF) of the percentage of driving time for (**a**) idling, (**b**) cruising, (**c**) accelerating, and (**d**) decelerating for the different times. Solid and dashed lines are for the 2016 and 2018 results, respectively.

The method used here provides the driving behaviour characteristics over seven time intervals per day, see “Materials and methods” section for more information. Figure 2 shows the probability distribution function (PDF) of the driving behaviours for the investigated time intervals. The solid and dashed lines here show the results for 2016 and 2018, respectively. The PDF profiles of the driving behaviour characteristics are similar across the two years studied, with a slight attenuation observed in the 2018 profiles compared to the 2016 profiles.

PDF profiles for idling driving and cruising move left and right, higher, and lower values as rush hour approaches. This is attributed to an increase in congestion, road use and therefore a decrease in average vehicle speed. Figure 2b shows that the cruising PDF profile is shifted further to the right (higher values) in 2016 (solid line) compared to 2018 (dashed line). Percentage time cruising (Pc) values in 2016 are on average 7.5% higher than in 2018. This could correspond to the increase in the number of cars in the studied years. Figures from the Department for Transport (DfT) show that the number of vehicles on the road in the UK increased by around 2.4% between 2016 and 2018. Accordingly, it can be expected that for the years under study, road use increased and thus percentage time cruising decreased.

**Figure 3:**
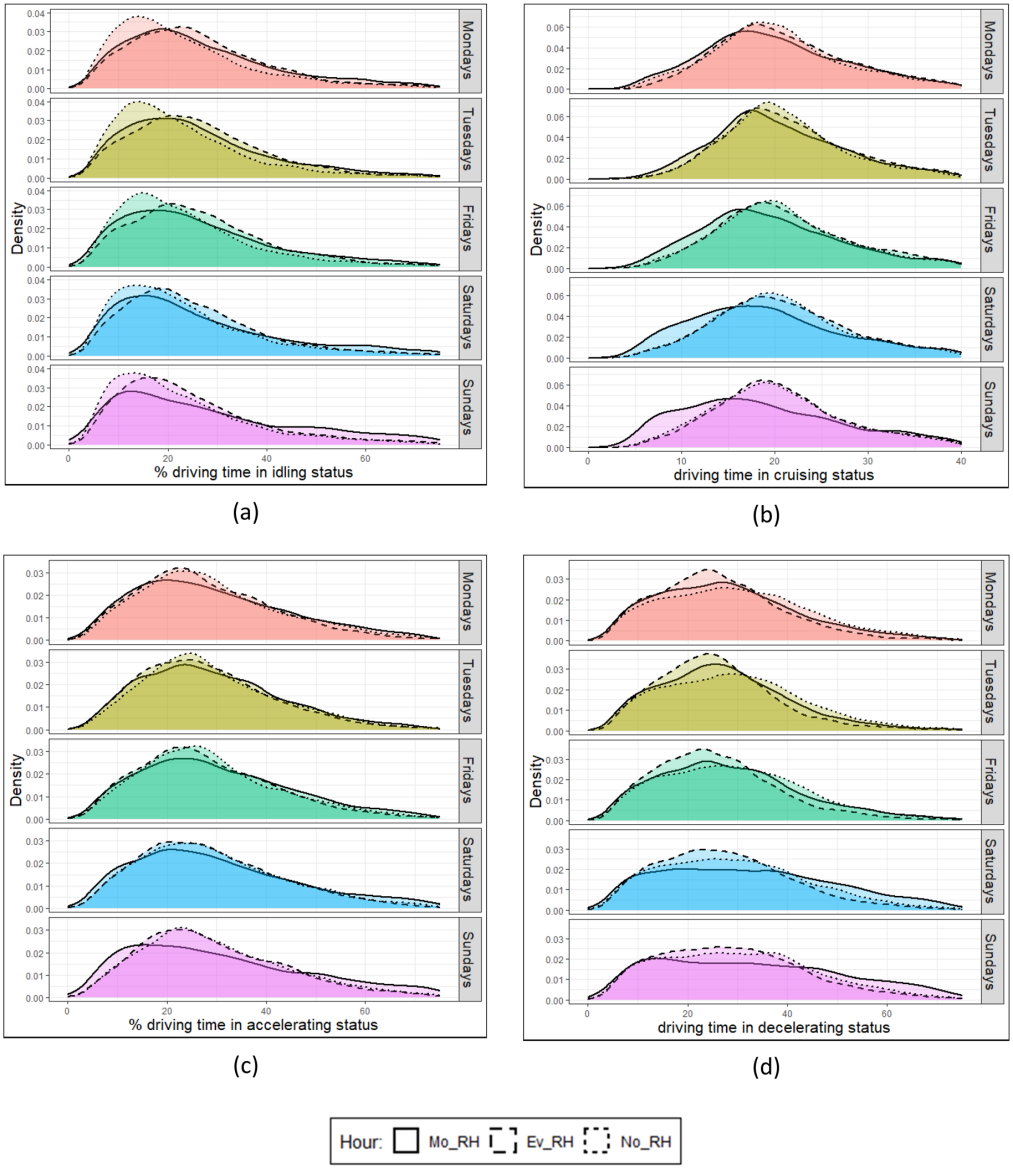
The probability distribution function (PDF) of the percentage of driving time spent for (**a**) idling, (**b**) cruising, (**c**) accelerating, and (**d**) decelerating for morning rush hours (Mo_RH), evening rush hours (Ev_RH), non-rush hours (No_RH) for the year 2016. Mo_RH, Ev_RH, and No_RH stand here for the hours 7–9, 16–19, and 14–16, respectively.

The PDF of driving behaviour characteristics over different days of the week is illustrated in Fig. 3. Solid, dashed, and dotted lines here correspond with the morning rush hours (Mo_RH), evening rush hours (Ev_RH), and non-rush hours (No_RH) for the year 2016. Mo_RH, Ev_RH, and No_RH represent the hours 07:00- 08:59, 16:00–18:59, and 14:00–15:59, respectively. Similar trends are observed for the 2018 profiles, and hence they are not shown.

Figure 3a shows the peak of the percentage time idling (Pi) PDF profile located at lower Pi values during non-rush hours compared to rush hours for all days of the week. The peak of the Pi PDF profile, especially for the morning rush hour, decreases as moving toward the weekends. Meanwhile, the Pi PDF largely maintains the same profile throughout the week. Figure 3b shows that the Pc PDF profile for evening rush hours and no-rush hours have almost identical weekday profiles. However, significant differences are observed in the Pc PDF profile for the morning rush hours. The unimodal variation of the Pc PDF profile decreases and becomes almost bimodal when moving to the weekends. Figure 3c shows that the percentage of driving time spent accelerating in the evening rush hour and non-rush hour is slightly higher than that in the morning rush hour for the whole week. Figure 3d shows that the percentage of driving time spent decelerating (slowing down) vehicles is somewhat higher in the evening rush hour than at other times during the week and at weekends.

**Figure 4:**
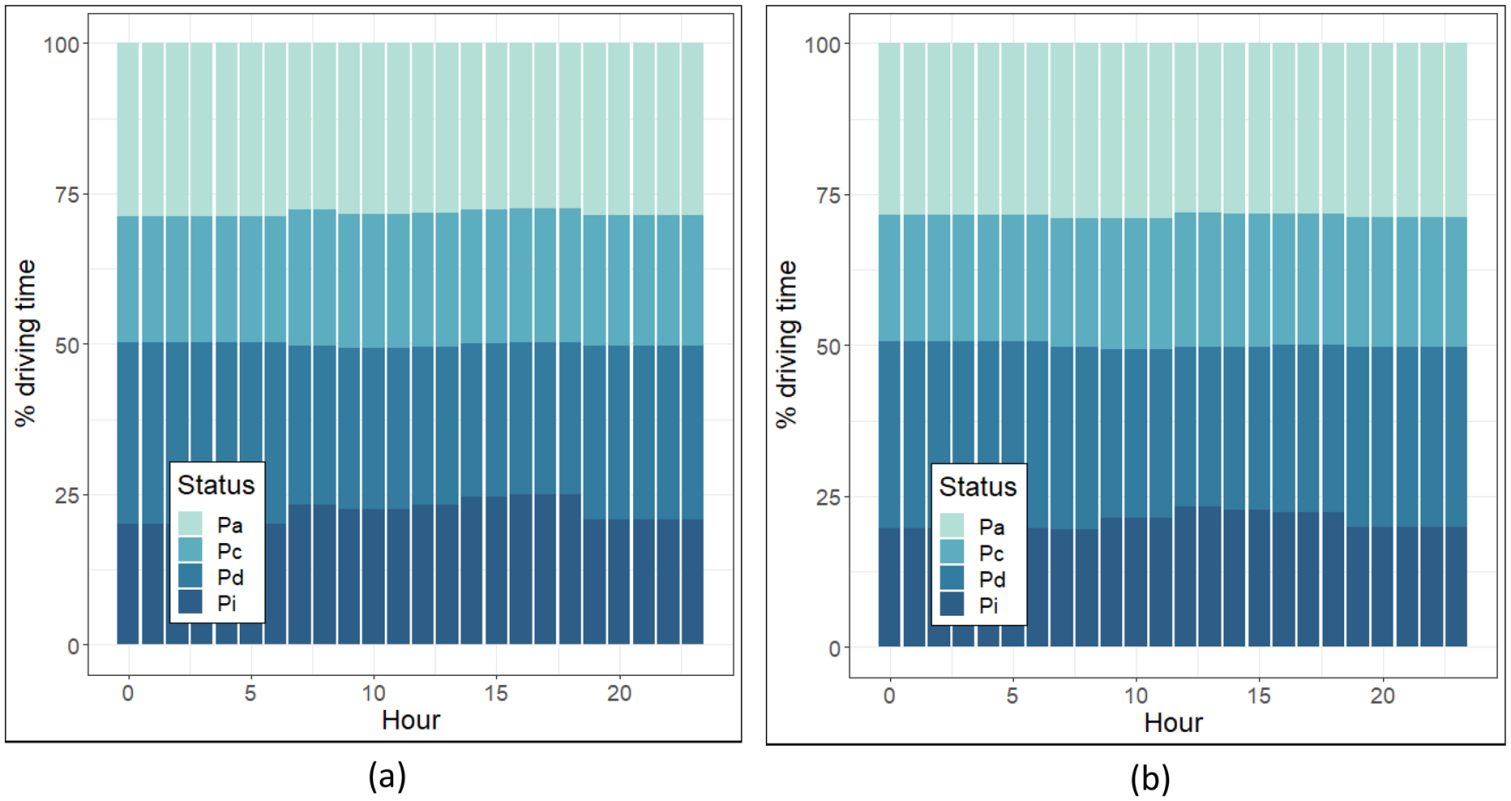
The hourly distribution of driving behaviour characteristics for (**a**) weekdays and (**b**) weekends in 2016, where Pa, Pc, Pd, and Pi stand for the percentage of driving time spent in acceleration, cruising, deceleration, and idling, respectively.

The hourly distributions of driving behaviour characteristics for weekends and weekdays in 2016 are shown in Fig. 4. Substantially similar changes were observed in the hourly distribution of the DB characteristics for 2018, so they are not shown here. Figure 4 shows the almost constant distribution of percentage time accelerating (Pa) and cruising (Pc) during the day. However, the distribution of Pi increases by about 5% in the afternoon, particularly during the evening rush hours, and then decreases by about 10% by moving toward late evening. Figure 4 shows an inverse variation between the distribution of Pi and Pd, where Pd increases as Pi decreases and vice versa.

### Driving volatility and aggressive driving over the urban environment

As mentioned above, there has been intense debate about how to detect aggressive driving. Extreme changes in acceleration have been widely used as a proxy for aggressive driving, while the non-linear relationship between acceleration and speed within real driving conditions has been largely ignored. Essentially, engines must work harder to maintain the same acceleration at higher speeds to overcome the increased drag forces. Therefore, a vehicle’s ability to accelerate or decelerate naturally decreases at higher speeds.

**Figure 5:**
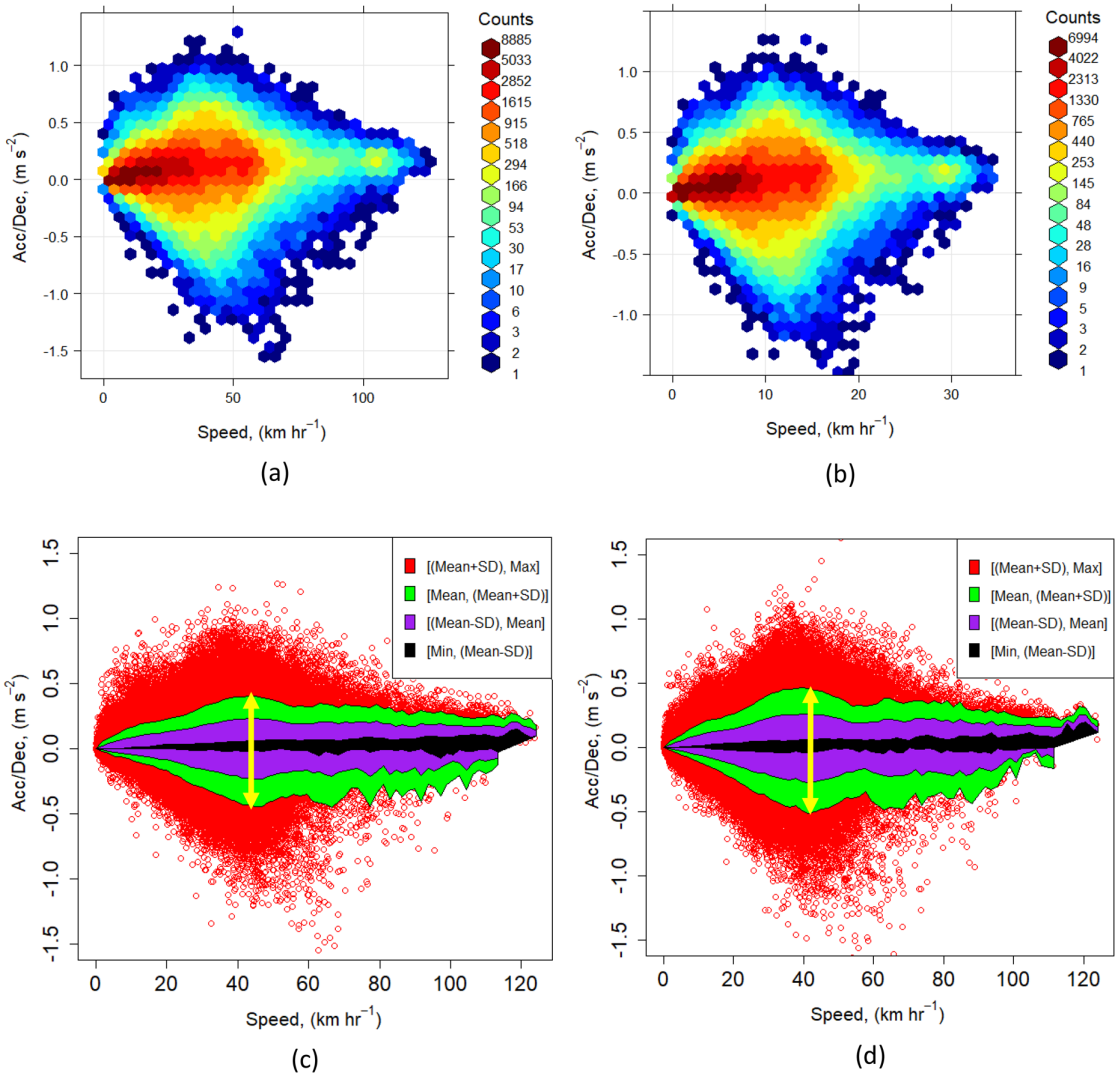
The speed-acceleration/deceleration distribution of GeoST segments in (**a**) 2016 and (**b**) 2018; the profile of speed versus acceleration/deceleration in (**c**) 2016 and (**d**) 2018.

We use the method of Wang et al.^7^, as discussed in the Material and Method section, to study the driving volatility in the studied segments. Figure 5 shows the speed and acceleration/deceleration of segments in the West Midlands for 2016 and 2018. In Fig. 5a,b, we show the profile distribution in terms of the corresponding segment counts, while Fig. 5c,d show the different regions of volatile driving behaviour for 2016 and 2018, respectively.

**Figure 6:**
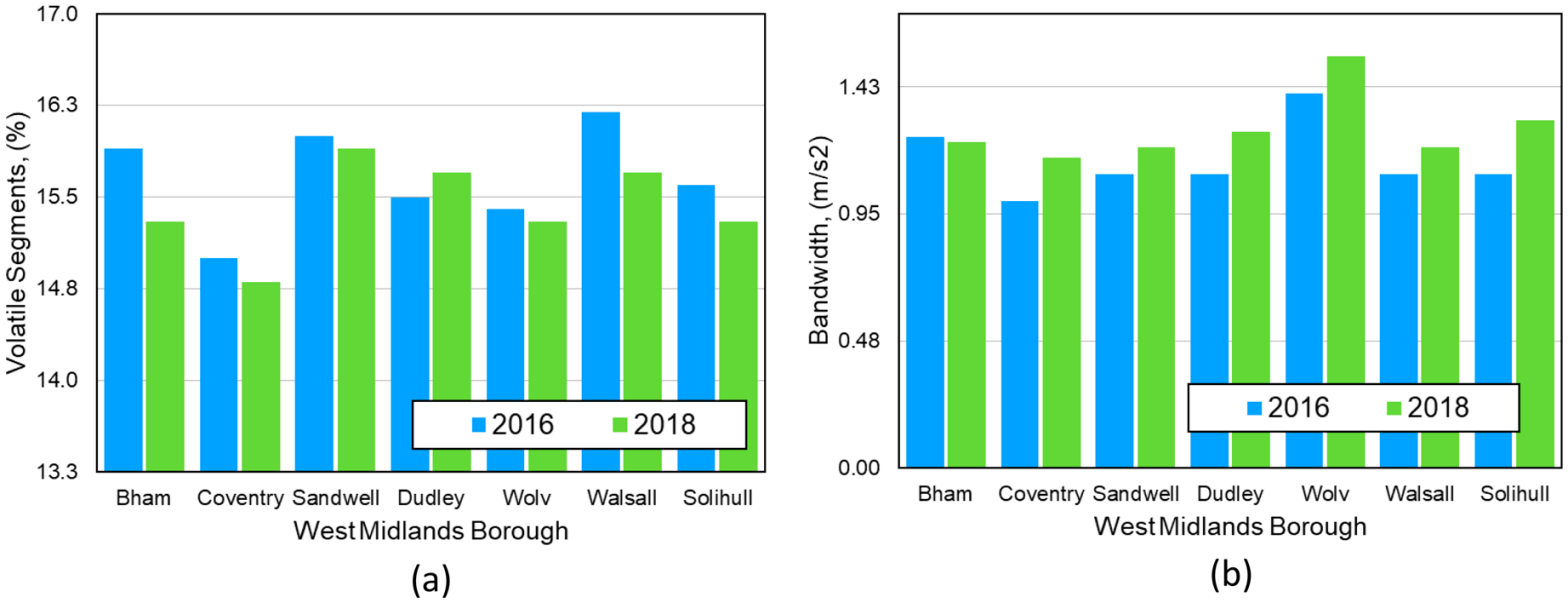
(**a**) The percentage of volatile segments and (**b**) the maximum bandwidth of the speed vs acceleration/deceleration profile of the West Midlands boroughs in 2016 and 2018. Birmingham is spelt ‘Bham’ and Wolverhampton is spelt ‘Wolv’ in the figures.

Using this profile, we can also classify the normal driving segments into segments with less, moderate, and very risky driving behaviours (outliers are volatile driving segments) based on their position relative to the central bell-shaped speed distribution. The distribution of the segment counts shown in Fig. 6a,b suggests an almost similar pattern of driving behaviour for the study years 2016 and 2018. The red dots (outliers) in Fig. 5c,d, are the segments with acceleration/deceleration values outside the bands of the mean plus/minus one standard division (SD). These segments are likely to have more potential for aggressive driving behaviours and can therefore be considered as volatile driving segments. In total, 15.6% and 15.2% of the studied segments for the years 2016 and 2018 are volatile ((15.8%, 14.8%) and (15.4%, and 14.7%) for (acceleration, deceleration)). In other words, the volatile segments (red dots) decreased by 2.6% between 2016 and 2018 most likely due to an increase in the number of cars. Figures from the UK Department for Transport (DfT) show that the number of registered cars in the country increased by 2.4% from 37.3 million in 2016 to 38.2 million in 2018^31,32^. This has the potential to increase the number of vehicles on the road, reduce the average speed of vehicles on the road and reduce the volatility of driving.

The bandwidth of the speed vs acceleration/deceleration profile, which is the difference between the upper and lower band values, provides insights into volatile driving. The bandwidth is determined in the direction of the acceleration/deceleration axis, so a decreasing or increasing bandwidth reflects decreasing or increasing speed change variation, respectively. In Fig. 5, the maximum bandwidths of the profiles are represented by double sided arrows.

We report the percentage of the volatile segments and the bandwidth of the West Midlands boroughs in Fig. 6 to examine the impact of regional sociodemographic factors such as population on driving volatility. The bar charts in Fig. 6 are ranked by population. In 2019, the population of the city of Birmingham (the most populous West Midlands borough) was more than 5.3 times that of the population of the city of Solihull (the least populous West Midlands borough). Figure 6 shows that the variation in percentage of volatile segments and maximum bandwidth for West Midlands boroughs is less than 10%.

**Figure 7:**
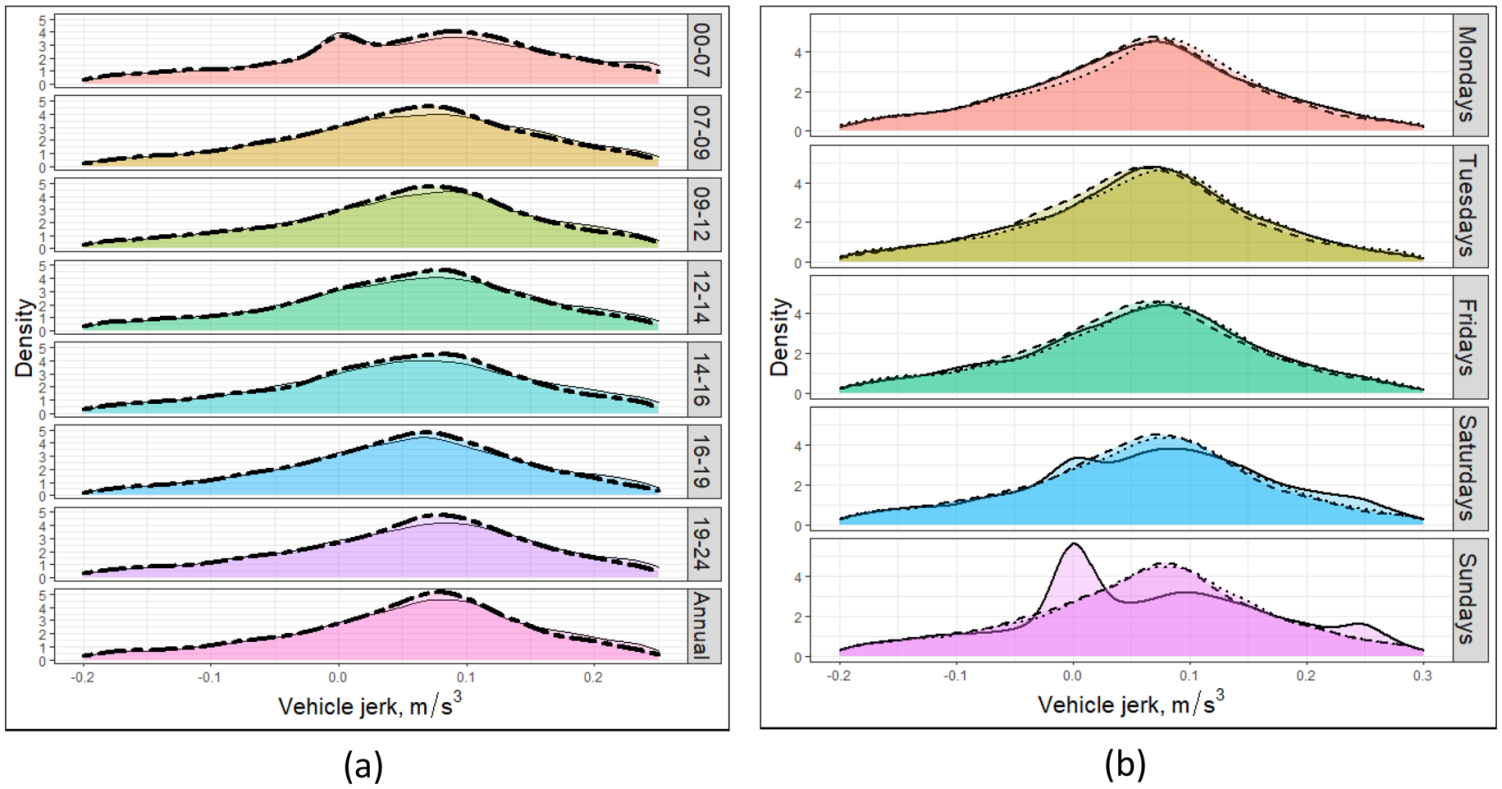
The (**a**) hourly and (**b**) daily variations of the probability distribution function (PDF) of vehicle jerk in the West Midlands. The solid and dashed lines in (**a**) represent the situation in 2018 and 2016 respectively, while the solid, dashed, and dotted lines in (**b**) are for the morning rush hours (Mo_RH), evening rush hours (Ev_RH), non-rush hours (No_RH) in 2018. Mo_RH, Ev_RH, and No_RH stand here for the hours 7–9, 16–19, and 14–16, respectively.

**Figure 8:**
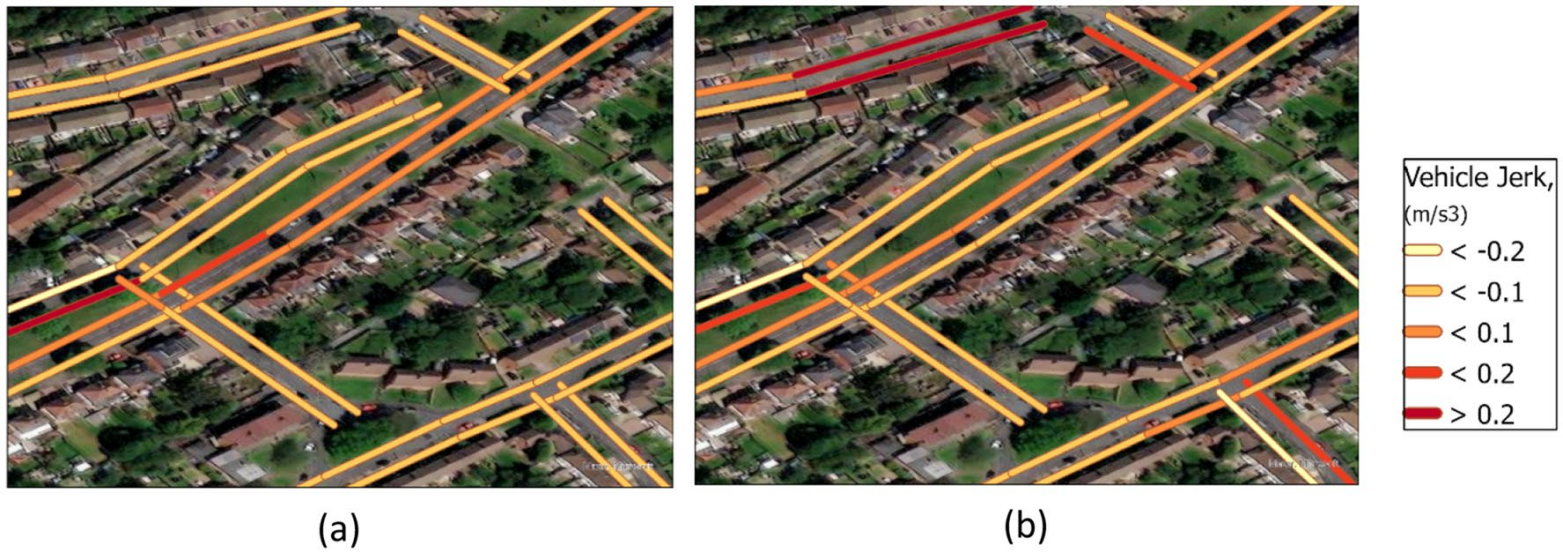
The spatial distribution of vehicle jerk in a small area of Birmingham, UK, for two periods on Mondays in 2018: (**a**) Morning rush hours 7–9 and (**b**) evening rush hours 16–19. The geographic features have been removed to avoid any potential impact on local affairs.

Vehicle jerk, as the second derivative of vehicle speed, is another proxy for aggressive driving. Figure 7a shows the hourly variation of vehicle jerk, with the solid and dashed lines representing the results for 2018 and 2016, respectively. It presents the variation of vehicle jerk across the entire West Midlands, providing a macro-scale analysis of the studied parameter. The PDF profile of vehicle jerk shows similar trends in both 2016 and 2018. However, the peak value in 2016 is slightly higher than that in 2018, indicating that aggressive driving behaviours were more prevalent in 2016 than in 2018. Except for the early morning (00:00–07:00), all other timeslots are unimodal and show the highest density with vehicle jerk from 0.05 to 0.10 m/s^3^, in the early morning, the PDF of vehicle jerk shows a bimodal, with peak values of density located at the 0.00 m/s^3^ and 0.10 m/s^3^. This study also allows for the examination of vehicle jerk and aggressive driving at both microscale and macros scales. Figure 8 provides an example of microscale variation in vehicle jerk during morning and evening rush hours on Mondays in 2018. The geographic attribution of the studied area has been removed to prevent potential conflicts with local affairs. The data demonstrates that vehicle jerk varies within the GeoST segments over time.

Figure 7b shows that on weekdays (Mondays, Tuesdays, and Fridays) the PDF profiles of vehicle jerks are almost the same between three different periods. However, on weekends (Saturdays and Sundays), the PDF profile of vehicle jerks for the morning peak period is almost tri-modal, while the other two periods (evening peak and off-peak) are unimodal. Compared to Saturdays, the shape of the tri-modal PDF profile for the morning peak period is more obvious on Sundays, the peak could be observed at 0.00 m/s^3^, 0.10 m/s^3^ and 0.25 m/s^3^ respectively, the peaks of the other two periods are in the range of 0.05 m/s^3^ to 0.10 m/s^3^ in all time slots. Therefore, it can be assumed that aggressive driving behaviour is more likely to occur during the morning rush hour on weekends than on weekdays and during the evening rush hour and off-peak hours on weekends. Note that the rush hour periods are defined by weekday driving times.

## Discussions

This study uses the novel approach of GeoSTMUM for the analysis of driving behaviour characteristics using telematics data with unprecedented spatial and temporal resolutions. By processing a large dataset of GPS-connected vehicles in the West Midlands, UK, the methodology successfully translates journey data into meaningful driving behaviour metrics across 354,000 GeoST-segments covering over 17,700 km of roads.

According to the probability distribution functions (PDF) of driving behaviour characteristics over different hours of a day and week, in West Midlands, the increased congestion during rush hours affects vehicle speeds, the PDF profiles for idling and cruising shift as rush hour approaches with higher idling percentages and lower cruising percentages. The percentage time cruising PDF profile in 2016 is 7.5% higher than 2018, possibly due to a 2.4% increase in UK vehicle numbers between these years. An examination of the PDF of driving behaviour characteristics over different days of the week shows consistent patterns during rush and non-rush hours. The peak of percentage time idling PDF profile is higher during non-rush hours and decreases toward weekends. The percentage time cruising PDF profile shows little variation during weekdays but changes during weekend mornings. The study also reveals close distributions in the range of 21–28% for all four studied driving behaviours (idling, accelerating, decelerating and cruising), while hourly changes do not appear to be significant.

The paper delves into the detection of aggressive driving, represented by extreme changes in acceleration and deceleration, and how this relationship can be nonlinear at higher speeds due to increased drag forces. Based on Wang et al.^7^, a method of categorising acceleration and deceleration into speed bins, divided into three behaviour regions (upper bands, lower bands, and middle bands) was employed. Driving events outside these bands were considered volatile, with results indicating that approximately 15% of driving time is classified as volatile.

The driving volatility was analysed for the West Midlands for 2016 and 2018. Segment counts in these years exhibited a similar pattern of driving behaviour, with segments falling outside the mean plus/minus one standard division (SD) considered as potentially aggressive or volatile. Figures showed a decrease in volatile segments from 15.6 to 15.2% between 2016 and 2018, likely corresponding to a rise in the number of cars on the road.

The bandwidth of the speed vs acceleration/deceleration profile, indicative of volatile driving, shows a correlation between the bell-shaped speed distribution and the bandwidth, suggesting drivers are less likely to accelerate or decelerate abruptly at high speeds. The study also examined the impact of sociodemographic factors on driving volatility, such as population, with less than 10% variation in volatile segments and maximum bandwidth across different West Midlands boroughs.

Vehicle jerk, the second derivative of speed, is another indicator of aggressive driving. Analysis of vehicle jerk showed a similar trend for both 2016 and 2018, except for a slightly higher peak value in 2016. However, aggressive driving behaviour was more likely to occur during morning rush hour on weekends than weekdays and evening rush hour, as shown by the tri-modal PDF profile of vehicle jerks on weekends. This highlights that aggressive driving behaviour does vary depending on the time and day of the week.

This paper presents a spatio-temporal analysis of driving behaviour and volatility at fine temporal (2-h) and spatial resolutions (15–150 m). We have demonstrated how this information can be used to generate a greater array of driving cycles using a segment-based approach for different spatio-temporal regions of interest. The methodology enhances our comprehension of driving behaviour and can be used to identify poor driving behaviour. Additionally, this information has the potential to incentivise good driving behaviour, thereby reducing emissions and traffic accidents associated with poor driving. Going forwards, the described method can be used in new approaches for road safety initiatives. Improvements in road safety can now hopefully be found by analysing the identified spatial and temporal patterns. New digital technologies, including digital twins of road traffic, the Internet of Things, vehicle-to-road communication, and signalised intersections, can all make use of the telematics derived data.

## Materials and methods

### Telematics data

Telematics data collected from urban road journeys of GPS-connected cars is the raw material used in this study. Journeys are speed-time data per destination pair, so the terms ‘telematics data’ and ‘urban journeys’ are henceforth used interchangeably. Telematics data was obtained from The Floow (www.thefloow.com), a UK telematics company that offers a wide range of insurance, mobility, and transport services. The Floow acquires the vehicle telematics data from a diverse array of UK-based insurance providers. Ghaffarpasand et al.^21^ discussed various methods for collecting telematics data, such as from drivers’ cell phones or on-board vehicle devices (OBD). The predominant source of data in this study results from OBD devices installed within cars, whose drivers aim to secure better insurance premiums. There are concerns that drivers who share their location data may exhibit cautious driving behaviour during monitoring, known as the Hawthorne effect^33^. However, research shows that this effect is temporary, with drivers reverting to their usual driving habits after 2–4 weeks^33^.

The data used here were collected from 3 to 7% of all cars on the road. Rates of GPS-connected cars vary by time and place of travel, for example, more connected vehicles travel on arterial roads during rush hours compared to that in suburban areas during non-rush hours.

The Floow performs thorough quality control and quality assurance on the collected data, then anonymises and aggregates it on geospatial and temporal (GeoST) road segments for specific times and days of the week. This anonymisation and aggregation step brings the data into compliance with EU and UK general data protection regulations (GDPR). The GeoST segments refer to specific road sections with distinct spatial and temporal attributes. The polylines, defined by specific latitude and longitude coordinates, covered all roads in the studied area. It is worth mentioning that the GeoST segments adhered to the standard national guidelines for road mapping in the United Kingdom (UK), as proposed in OpenStreetMap (https://wiki.openstreetmap.org/wiki/Roads_in_the_United_Kingdom). The Floow supplies this study by delivering speed acceleration frequency distribution (SAFD) matrix for each GeoST segment in the studied area. The definition of the SAFD matrix can be found in the following sections.

### Scope of the study

Telematics data are collected from West Midlands roads in the UK for 2016 and 2018. The West Midlands (WM) is a county located in the centre of England in the UK, with a population of over 3 million in 2019. The WM region consists of seven boroughs: Birmingham, Coventry, Dudley, Sandwell, Solihull, Walsall, and Wolverhampton. With a total road length of around 17,700 km, WM roads represent approximately 4.43% of all roads in England^34^. According to national road statistics, the number of on-road cars in WM increased from about 2.85 to 3.06 million for the five years from 2014 to 2019. Fleet composition and the contribution of petrol and diesel cars are reported by Osei et al.^35^. A report from the UK Department for Transport (DfT) shows that annual traffic in terms of cars and taxis-miles travelled on WM roads increased by 2% between 2016 and 2018^36^. Meanwhile, the annual traffic in miles travelled on different roads has been almost constant for the years under study, with 38.5%, 36.4%, and 25.1% of the miles travelled being allocated to minor roads, A-roads, and motorways, respectively^36^; see^34^ for the definition of different road types across the UK. Therefore, it can be concluded that the distribution of traffic flow on WM roads was stable for the studied periods of 2016 and 2018.

### GeoSTMUM and intuitive vision of driving behaviour

The telematics data collected as successful journeys between each pair of destinations are translated into several road transport attributes using the geospatial and temporal mapping of urban mobility (GeoSTMUM) approach recently developed by Ghaffarpasand and Pope^37^. GeoSTMUM has previously been used to study traffic dimensions, traffic noise^3^, vehicle emission and fuel consumption^10^, while in this study we use it to analyse the driving behaviour (DB) characteristics in urban environments.

GeoSTMUM involves aggregating the speed-time data of all journeys made on specific GeoST segments. GeoST-segments vary in length from 15 to 150 m, have specific geospatial coordinates for each pair of starting and ending points. They were designed in accordance with traffic flow, whereby a pair of cross GeoST-segments in the upstream and downstream direction of traffic was determined for each piece of the road except one-way streets. 353,579 GeoST-segments cover the entire West Midlands road network, while only 12.3% of the segments were located on motorways, trunk, primary, secondary, and tertiary roads, and the rest were located on other road types, such as residential, service links, around the roundabouts, etc.

A temporal identity was also assigned to each GeoST-segment. They provide road transport status for 35-time intervals (each in 2016 and 2018), seven time intervals a day including 00:00–06:59, 07:00–08:59, 09:00–11:59, 12:00–13:59, 14:00–15:59, 16:00–18:59, and 19:00–23:59, for 5 days a week (Mondays, Tuesdays, Fridays, Saturdays, and Sundays). This temporal resolution provides the opportunity to study the factor of time of travel, particularly non-rush/rush hours and weekdays/weekends effects on driving behaviour characteristics.

The aggregated speed-time data (urban journeys) in each GeoST-segment is then used to examine the speed-acceleration-frequency-distribution (SAFD) matrix. SAFD provides the distribution of the speed-time data in the certain speed-acceleration bins, see Ghaffarpasand and Pope^37^ for more details. Driving behaviour characteristics are then estimated for every GeoST-segment.

Driving behaviour is typically characterized by the distribution of idling, cruising, accelerating, or decelerating statuses, which are defined by the following criteria^38^:

1 $$\begin{array}{*{20}l} {\text{Idling}}:{\text{ v }} \le {\text{ 5 km}}/{\text{h}}, \hfill \\ {\text{Acceleration}}:{\text{ v }} > {\text{ 5 km}}/{\text{h}},{\text{ and a }} > \, 0.{\text{1 m}}/{\text{s}}^{{2}} , \hfill \\ {\text{Decceleration}}:{\text{ v }} > {\text{ 5 km}}/{\text{h}},{\text{ and a }} < \, - 0.{\text{1 m}}/{\text{s}}^{{2}} , \hfill \\ {\text{Cruising}}: \, - 0.{\text{1 m}}/{\text{s}}^{{2}} \le {\text{ a }} \le \, 0.{\text{1 m}}/{\text{s}}^{{2}} . \hfill \\ \end{array}$$

These criteria have been used by many previous investigators such as Saleh et al.^39^, Amirjamshidi and Roorda^40^, and Huang et al.^41^ to study the driving dynamics of urban environments. We implemented them on SAFDs to estimate the distribution of idling, acceleration, deceleration, and cruising statuses for each GeoST-segment, denoted here by ${P}_{i}$, ${P}_{a}$, ${P}_{d}$, and ${P}_{c}$, respectively. Driving time is also calculated by the ratio of segment length and average speed for each GeoST-segment.

The GeoST-segments are not equally weighted in terms of annual traffic volume. We used the method presented by Ref.^30^ to assign a specific weight to each GeoST-segment. Liu et al.^30^ used the traffic volume data to classify roads into low, medium, and high-speed phases with thresholds of 60 km/h and 80 km/h. Then the weighting factors are estimated by the ratio of the traffic volume ratio of each speed phase to the total annual traffic volume. We used the West Midlands traffic volume map for 2016 obtained from Birmingham City Council (BCC), which shows the contributions of 81.2%, 12.9%, and 5.9% for low, medium, and high-speed phases, respectively; the total annual traffic volume was more than 1.9e+08. We used a similar distribution for 2018 as the UK Department for Transport (DfT) report, which indicated that all dimensions of traffic for West Midlands increased by only around 2% in 2016–2018^42^.

We applied a series of exclusion criteria to exclude uncertain GeoST-segments from calculations. Accordingly, we only looked through motorways, trunk, primary, secondary, and tertiary roads, and excluded all GeoST-segments located in residential areas, service links, around roundabouts, intersections, junctions, etc. We also excluded the GeoST-segments with idling periods longer than 200 s and shorter than 5 s. We also eliminated segments with an average speed higher than 130 km/h or less than 3.6 km/h. Such exclusions were implemented by previous investigators such as Liu et al.^30^. After this exclusion and taking into account the assigned weights, the averaged driving characteristics are then examined.

### Aggressive driving and travel volatility

Aggressive driving occurs as a result of rapid and sudden driving decisions. Vehicle jerk, which is the temporal derivative of acceleration, is typically used to gauge passenger preference. A range of − 0.9 to 0.9 m/s^3^ has been identified as the ideal passenger preference during normal driving^15,43^. Nonetheless, Wang et al.^7^ estimated the vehicle jerk during a specific journey and then scrutinised the aggressive driving behaviour. The vehicle jerk for a typical journey is given by Wang et al.^7^:

2 $$J= \frac{da(t)}{dt}=\frac{dv(t)}{dt}\times \frac{da(t)}{dv(t)}=a\left(t\right)\times \frac{da(t)}{dv(t)}.$$

The range of vehicle jerk over the journeys studied by Wang et al.^7^ was in the range of − 0.3 to 0.4 m/s^3^. In this study we estimate the vehicle jerk over the GeoST-segments to provide an accurate spatial and temporal distribution of travel volatility within the urban environment.

Most available research studies used time-varying acceleration to discuss aggressive driving. Hence, the complex and non-linear relationship between acceleration and speed, known as driving dynamics, is difficult to assess. In this study, we look at the acceleration/deceleration profile and average speed to deepen the existing understanding of aggressive driving and volatile driving within urban environments. Acceleration and deceleration statuses are determined using Eq. (1).

To analyse driving volatility, Wang et al.^7^ examined the speed and acceleration/deceleration characteristics during urban travel and then assessed normal or aggressive driving. Acceleration and deceleration are identified with positive and negative acceleration values, respectively. Acceleration/deceleration was then grouped into specific speed bins. Finally, they divided the speed and acceleration/deceleration profile into three driving behaviour regions: (i) upper bands, which are distributed between the mean acceleration/deceleration and the mean plus one standard deviation, (ii) lower bands, which are distributed from the mean acceleration/deceleration and the mean minus one standard deviation, and (iii) the middle band distributed from minimum acceleration/deceleration and the mean minus one standard deviation. According to Wang et al.^7^, driving events within and outside these bands were considered as typical driving practices and volatile driving, respectively. They found that 5.0% of driving could be classified as volatile, 15.7% for acceleration and 14.5% for deceleration. We use the same approach to discuss the volatility of driving on the roads of the West Midlands.

## Data Availability

The data that support the findings of this study are available from the corresponding author, Prof. Francis D. Pope, upon reasonable request.
